# Spinal Cord Injury in Myelomeningocele: Prospects for Therapy

**DOI:** 10.3389/fncel.2020.00201

**Published:** 2020-06-30

**Authors:** Karolina Janik, Meredith A. Manire, George M. Smith, Barbara Krynska

**Affiliations:** ^1^Shriners Hospitals Pediatric Research Center, Center for Neural Repair and Rehabilitation, Lewis Katz School of Medicine at Temple University, Philadelphia, PA, United States; ^2^Department of Obstetrics and Gynecology, West Penn Hospital, Allegheny Health Network, Pittsburgh, PA, United States

**Keywords:** myelomeningocele, spina bifida, neural tube defects, spinal cord injury, neural injury

## Abstract

Myelomeningocele (MMC) is the most common congenital defect of the central nervous system and results in devastating and lifelong disability. In MMC, the initial failure of neural tube closure early in gestation is followed by a progressive prenatal injury to the exposed spinal cord, which contributes to the deterioration of neurological function in fetuses. Prenatal strategies to control the spinal cord injury offer an appealing therapeutic approach to improve neurological function, although the definitive pathophysiological mechanisms of injury remain to be fully elucidated. A better understanding of these mechanisms at the cellular and molecular level is of paramount importance for the development of targeted prenatal MMC therapies to minimize or eliminate the effects of the injury and improve neurological function. In this review article, we discuss the pathological development of MMC with a focus on *in utero* injury to the exposed spinal cord. We emphasize the need for a better understanding of the causative factors in MMC spinal cord injury, pathophysiological alterations associated with the injury, and cellular and molecular mechanisms by which these alterations are induced.

## General Characteristics and Pathology of MMC

**Figure 1:**
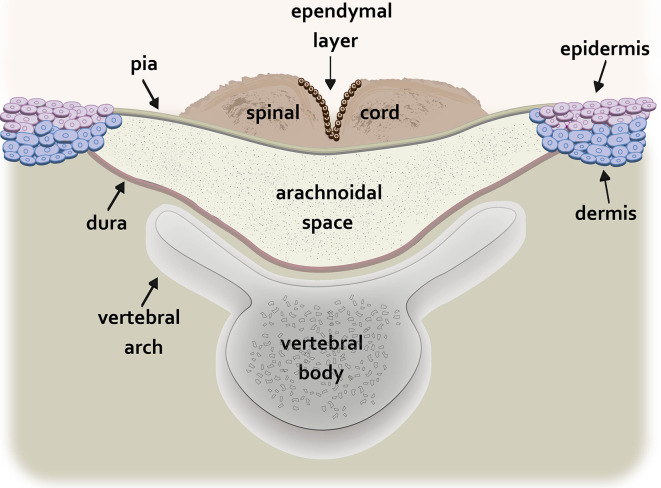
Schematic drawing of the transverse spinal cord at the level of the myelomeningocele (MMC) lesion. The non-neurulated spinal cord is visible on the dorsal aspect of a fluid-filled sac containing cerebrospinal fluid. The cystic sac is formed dorsally by the pia and ventrally by the dura. The edges of the pia are fused to the epidermis and superficial dermis while the dura is fused to the deeper dermal layers. The vertebral arch of the spinal column is incompletely formed.

Myelomeningocele (MMC), the most common and severe form of spina bifida, is a complex congenital defect that results from incomplete neural tube closure (Copp et al., 2003). The defect is characterized by protrusion of the spinal cord and meninges through a pathological opening in the overlying vertebrae and skin leading to progressive injury to the exposed spinal cord associated with devastating and lifelong disabilities (Kaufman, 2004). In humans, the MMC defect is typically found in the lumbosacral region (Borgstedt-Bakke et al., 2017; Farmer et al., 2018). The non-neurulated placode-like spinal cord is exposed on the dorsal aspect of a cystic sac containing cerebrospinal fluid bordered by the pial and dural membranes (Figure 1; Hutchins et al., 1996).

In addition to spinal cord injury, MMC predisposes the fetus to hindbrain herniation and the development of Arnold-Chiari II malformation, with consequent hydrocephalus requiring ventriculoperitoneal shunt placement (Kaufman, 2004). Patients living with MMC experience substantial and life-long deficits, including reduced or absent motor and sensory function in the lower extremities, musculoskeletal deformities, bowel and bladder dysfunction, and cognitive disabilities (Hunt, 1990; Dias and McLone, 1993; Hunt and Poulton, 1995; Tomlinson and Sugarman, 1995; Hunt and Oakeshott, 2003). Moreover, many patients affected by MMC develop secondary complications, such as urinary tract infections, chronic renal disease, ventriculoperitoneal shunt failure, and various skin injuries (e.g., pressure ulcers in those who are non-ambulatory; Verhoef et al., 2004; Dicianno and Wilson, 2010). Affected individuals often require multidisciplinary medical services to optimize functional outcomes and lifelong caregiver support. Ultimately, MMC not only diminishes the quality of life but results in social, emotional, and financial burden on the afflicted families and the health care system (Bowman et al., 2001; Centers for Disease Control and Prevention (CDC), 2007; Sandler, 2010; Radcliff et al., 2012).

While the exact etiology of MMC remains poorly understood, the development of MMC is likely the result of both environmental factors and genetic aberrations (Alles and Sulik, 1990; Mitchell et al., 2004; Kibar et al., 2007; Ornoy, 2009). It is generally accepted that inadequate intake of natural folate or folic acid is associated with an increased risk of neural tube defects, including MMC (Botto et al., 1999; Imbard et al., 2013). Therefore, folate intake, either from dietary supplements or fortified food, is recommended for every reproductive age woman (Viswanathan et al., 2017). Although these strategies have helped to decrease the number of neural tube defects, with some variability between countries or ethnic groups (Heseker et al., 2009; Williams et al., 2015), MMC still affects approximately 3 per 10,000 live births in the United States (Parker et al., 2010; Canfield et al., 2014). Overall, neural tube defects remain among the most common major congenital abnormalities in humans and constitute a substantial public health problem (Oakley, 2010; Parker et al., 2010; Osterhues et al., 2013; Moldenhauer, 2014).

## Spinal Cord Injury in MMC

A “two-hit hypothesis” has been proposed to explain the pathogenesis of spinal cord injury and neurological sequelae in MMC (Heffez et al., 1990; Meuli et al., 1995a; Hutchins et al., 1996). The defect in neurulation, which results in incomplete closure of the developing neural tube during early gestation, represents the first hit. This leads to spinal cord malformation (non-neurulation) and its subsequent protrusion through the pathological opening in the overlying vertebrae, musculature, and skin. The chronic exposure of the unprotected spinal cord to the hostile intrauterine environment throughout gestation represents the second hit. This results in an acquired injury to the exposed segment of the non-neurulated spinal cord.

Compelling clinical and experimental evidence indicates that the neurological deficits associated with MMC are not caused entirely by the primary defect of neurulation but are exacerbated by *in utero* spinal cord injury (Heffez et al., 1990; Meuli et al., 1995b; Sival et al., 1997; Stiefel et al., 2007). Histopathological data from human specimens reveal that the exposed part of the non-neurulated spinal cord is histologically intact in early gestation, however, progressive damage showing signs of degeneration, abrasion, and erosion—sometimes leading to an almost complete loss of neural tissue in the dorsal region—is found by late gestational age (Hutchins et al., 1996; Meuli et al., 1997). Also, sonographic reports of human fetuses with MMC show progressive loss of lower extremity movements, suggesting that prolonged and ongoing exposure to the intrauterine environment is associated with the functional loss (Korenromp et al., 1986; Sival et al., 1997). Experimental data obtained from various animal models of MMC further support the hypothesis that exposure of the MMC spinal cord to the intrauterine environment leads to chronic injury and deterioration of neural function. Studies of mouse genetic models show that in fetuses affected by MMC, spinal cord tissue taken from early gestational time points has no overt evidence of trauma or degeneration and the spinal cord cytoarchitecture, including sensory and motor projections, appear to be well-preserved (Selçuki et al., 2001; Stiefel and Meuli, 2007; Stiefel et al., 2007). As gestation proceeds, there is increasing damage to the exposed spinal cord which coincides with a gradual loss of neurological function (Stiefel et al., 2007). Other experimental data using a retinoic acid-induced model of MMC in fetal rats also demonstrate the structural deterioration of the exposed spinal cord with advancing gestational age, associated with a loss of functional capacity that is present earlier in pregnancy (Danzer et al., 2011). Furthermore, evidence from rat, pig, rabbit, and sheep models in which MMC is surgically created shows that direct exposure of the normal spinal cord to the intrauterine milieu leads to MMC-like lesions and functional loss similar to that observed in human MMC. The spinal cord destruction observed during pregnancy is progressive, with later gestational age fetuses exhibiting more severe damage than those examined at earlier time points (Heffez et al., 1990, 1993; Meuli et al., 1995a,b, 1996; Housley et al., 2000).

In addition to the described structural and functional evidence, injury to the exposed MMC spinal cord is also demonstrated through the examination of astrogliosis. Astrogliosis is a common feature of CNS injury and is associated with increased glial fibrillary acidic protein (GFAP) immunoreactivity (Buffo et al., 2008; Robel et al., 2011; Yu et al., 2012). In studies using a genetic mouse model of MMC, an enhancement in astrocyte density is observed in sections of the spinal cord taken from the MMC lesion site of late gestational age fetuses (Reis et al., 2007). In separate studies, using a retinoic acid-induced rat model of MMC, Danzer et al. (2011) showed that injury upregulates the number of GFAP-expressing cells in the dorsal spinal cord as gestation proceeds. Increased GFAP staining and other markers of inflammatory infiltrate were also identified in resected human postnatal placode tissue (George and Cummings, 2003; Kowitzke et al., 2016). Pathophysiology of spinal cord injury in MMC was also evaluated using transcriptomic analysis of human amniotic fluid samples that showed differentially regulated genes commonly associated with inflammation and neuronal development (Tarui et al., 2017). Taken together, these studies support the hypothesis that *in utero* spinal cord injury by way of ongoing pathophysiological changes is an antecedent to loss of neurological function. However, cellular and molecular mechanisms underlying the formation of astrocytosis as well as other pathophysiological derangements that parallel the injury have not yet been entirely defined.

## Multifactorial Etiology of Spinal Cord Injury

While the mechanisms underlying prenatal injury to the exposed spinal cord remain to be fully elucidated, human and animal studies indicate that the intrauterine environment contributes substantially to spinal cord damage in MMC. It was postulated that chemical and/or mechanical insults to the unprotected fetal spinal cord abnormally exposed to the amniotic fluid and the uterine wall are involved in the progressive *in utero* damage (Heffez et al., 1990, 1993; Meuli et al., 1995a,b). Toxic injury caused by components of the amniotic fluid may constitute one of the contributing mechanisms. The presence of fetal urine and meconium were indicated as possible factors for the toxicity of late gestational amniotic fluid (Heffez et al., 1990; Meuli et al., 1995a; Correia-Pinto et al., 2002). In the i*n vitro* assessment of amniotic fluid, toxicity has been primarily based on the assay for lactate dehydrogenase efflux in organotypic rat spinal cord cultures following treatment with human amniotic fluid. Despite that higher activity of this enzyme is detected after treatment of cultures with late gestational amniotic fluid, this study showed no confirmatory evidence of tissue damage (Drewek et al., 1997). Other *in* vitro experiments evaluating the toxicity of α-amylase, a component of meconium, indicate that survival of neuroepithelial cells isolated from rat embryos appears to decrease when this digestive enzyme is added to cell culture. However, the actual concentration and activity of α-amylase in the MMC amniotic fluid were not established (Sasso et al., 2020). Interestingly, several recent studies have identified a multitude of live astrocytes and neural stem cells within the AF with amniotic fluid of MMC fetuses, indicating little, if any, neurotoxicity (Pennington et al., 2013, 2015; Turner et al., 2013; Zieba et al., 2017). Also, studies using fetal lambs reported that MMC promoted mesenchymal stem/progenitor cell phenotypes and proliferation in the amniotic fluid (Ceccarelli et al., 2015), suggesting that amniotic fluid in MMC may be growth permissive.

As mentioned above, another postulated mechanism contributing to spinal cord damage in MMC includes mechanical trauma to the herniated spinal cord (Heffez et al., 1990, 1993; Meuli et al., 1995a,b). This mechanism may be more relevant in the later stages of gestation when the volume of amniotic fluid naturally begins to decline. Without protection by the amniotic fluid, there may be higher abrasive stress and shear forces exerted by the uterine wall onto the exposed neural tissue. Pathological specimens of human MMC do demonstrate signs of abrasion and loss of neural tissue almost exclusively in the dorsal spinal cord at the site of MMC defect, supporting the involvement of mechanical trauma in neural injury (Hutchins et al., 1996; Meuli et al., 1997; Sutton et al., 2001). Dorsal displacement of the spinal cord and the emergence of neural stem cells within the amniotic fluid during the development of rat MMC fetuses is also consistent with the contribution of mechanical trauma to neural damage (Danzer et al., 2011; Zieba et al., 2017). Our recent studies show that these pathological changes coincide with decreased levels of hyaluronic acid and lower amniotic fluid viscosity of late gestational age rat MMC fetuses (Zieba et al., 2019). We hypothesized that decreased viscosity of amniotic fluid surrounding MMC fetuses causes reduced lubrication between the exposed spinal cord and amniotic sac, further increasing abrasive or shearing stress on the exposed spinal cord (Zieba et al., 2019). Recently, Oliver et al. (2020) suggested that hydrostatic forces exerted on the exposed neural placode and nerve roots by cerebrospinal fluid may cause additional neurologic injury in MMC through a stretching or traction mechanism. Altogether, these data emphasize the multifactorial etiology of prenatal spinal cord injury and the need for a deeper understanding of the causative factors and pathophysiology of injury in MMC-afflicted fetuses.

## Postnatal and Prenatal Repair for MMC

Traditionally, the spinal cord defect in MMC has been surgically closed soon after birth. Early postnatal repair may reduce infection or trauma to the spinal cord but affected neonates will still require life-long support, rehabilitation, or institutional care (Akalan, 2011; Perin et al., 2008; Roach et al., 2011). Mounting clinical and experimental evidence showing neurological deterioration and progressive damage to the open spinal cord during gestation provided the rationale for exploring prenatal surgical repair strategies (Korenromp et al., 1986; Heffez et al., 1990; Meuli et al., 1995b, 1997; Hutchins et al., 1996; Sival et al., 1997; Stiefel et al., 2007). This idea was further supported by animal studies demonstrating that *in utero* repair of surgically created MMC defects improved functional outcomes, including motor and sensory function, and prevented hindbrain herniation (Heffez et al., 1993; Meuli et al., 1995a, 1996; Paek et al., 2000). With the improvement of prenatal surgical techniques and prenatal detection of neural tube defects, *in utero* open repair of MMC lesions in humans has become a viable strategy to minimize spinal cord trauma and lessen neurological decline before birth (Adzick, 2012). A multicenter randomized prospective trial comparing outcomes of *in utero* fetal repair with postnatal surgical repair of MMC defects demonstrated a reduced number of children with hindbrain herniation, decreased need for cerebrospinal fluid shunting, as well as an increase in the ability to walk independently at 30 months of age in the prenatal surgery group (Adzick et al., 2011; Simpson and Greene, 2011). There were, however, considerable maternal and fetal risks, such as increased incidence of chorioamniotic membrane separation, spontaneous rupture of amniotic membranes, and preterm delivery in the prenatal surgery group (Adzick et al., 2011; Simpson and Greene, 2011; Johnson et al., 2016). Analysis of the full cohort data of 30-month outcomes did validate *in utero* open surgical repair as a treatment option for fetuses with MMC (Farmer et al., 2018). Follow-up cohort studies of school-age children showed improvement in mobility and fewer surgeries related to shunt placement or revision in those children who underwent prenatal surgery for MMC over those in the postnatal surgery group (Houtrow et al., 2020). While *in utero* open surgical MMC repair is neither a cure nor free of risk, prenatal repair is more effective than postnatal repair and is now a treatment option for patients who meet inclusion criteria (Adzick et al., 2011; Meuli and Moehrlen, 2014; Moise et al., 2016; Moldenhauer and Flake, 2019; Danzer et al., 2020).

In addition to open fetal surgery for MMC repair, minimally invasive fetoscopic surgery and closure techniques are currently being optimized. These less invasive approaches may reduce the maternal or fetal risk associated with open repair and can be applied earlier in gestation (Belfort et al., 2017; Joyeux et al., 2018). Despite these advances, closure of the MMC defect alone may not be sufficient to repair or prevent spinal cord damage in MMC, hence, additional strategies are necessary to promote functional recovery (Blumenfeld and Belfort, 2018).

Tissue engineering has recently emerged in experimental studies as a potential therapeutic approach to further improve outcomes of prenatal MMC repair (Watanabe et al., 2010, 2011, 2015, 2016; Dionigi et al., 2015a,b; Feng et al., 2016; Mazzone et al., 2016). Studies attempting to protect the spinal cord from intrauterine trauma by using tissue scaffolds, with or without stem cells, or intraamniotic delivery of stem cells demonstrate variable, but some improvement in outcome measures (Watanabe et al., 2010, 2011, 2015, 2016; Dionigi et al., 2015a,b; Feng et al., 2016). Continuing advances in our understanding of the pathological development of MMC defects, as well as the cellular and molecular mechanisms underlying the pathophysiology of spinal cord injury, are needed to develop novel treatment approaches so that we may more effectively intervene.

## Perspective

MMC is a devastating congenital defect that results from incomplete neural tube closure early in gestation followed by exposure of the affected spinal cord segment to the intrauterine environment. It is well established that this leads to prenatal spinal injury associated with deterioration of neural function (Korenromp et al., 1986; Hutchins et al., 1996; Meuli et al., 1997; Sival et al., 1997; Stiefel et al., 2007). Although the defect in neural tube closure is currently irreversible, eliminating or minimizing prenatal injury to the persistently exposed spinal cord is a promising therapeutic approach to improve neurological outcomes. Therefore, MMC studies have focused on optimizing closure of the MMC defect to protect the spinal cord from ongoing intrauterine trauma using surgical and/or tissue engineering approaches (Watanabe et al., 2015; Botelho et al., 2017; Danzer et al., 2020; Lazow and Fauza, 2020). To further improve neurological outcomes, development of targeted prenatal therapies must be an essential direction of MMC research. However, this requires a better understanding of pathophysiological mechanisms underlying the spinal cord injury.

Prior experimental and clinical MMC studies demonstrate the susceptibility of the exposed spinal cord to mechanical and/or chemical trauma within the intrauterine environment (Heffez et al., 1990, 1993; Meuli et al., 1995a,b, 1997; Hutchins et al., 1996). While *in utero* damage to the exposed spinal cord is well established, factors that trigger and/or contribute to the progression of the injury are not fully understood. Due to the dynamic nature of fetal development and *in utero* environment, these factors may also vary at different stages of gestation. Further investigation of contributory elements and more mechanistic studies are necessary to further elucidate the mechanisms of injury.

Identification of pathological alterations that parallel the injury in the unprotected segment of the developing MMC spinal cord is particularly important for pathophysiological understanding. This includes, but is not limited to, a full comprehension of the cellular and molecular mechanisms leading to the formation of astrocytosis observed at the MMC lesion site. In addition to architectural disruption, abnormal accumulation of astrocytes within the injured spinal cord tissue could be detrimental to spinal cord development in MMC fetuses.

Additionally, abnormalities in the development of neurons and their innervation patterns in MMC would greatly compromise the recovery. To date, very few studies have examined changes in the neural development of the spinal cord at the MMC site (Keller-Peck and Mullen, 1997; George and Cummings, 2003; Reis et al., 2007). Disruption of normal dorsoventral patterning due to the MMC lesion could lead to changes in numbers and distribution of developing neurons that establish vital local circuits involved in sensory and motor functions. As a systematic analysis of spinal cord development is not possible in human tissue, MMC experimental models could fill this gap and greatly influence future therapies.

## Conclusions

While innovative *in utero* surgical techniques exemplify the substantial progress which has been made in the diagnosis and treatment of MMC, continued advancement in the field of MMC repair requires a deeper understanding of the pathophysiology of spinal cord injury. By elucidating the cellular and molecular mechanisms of neurological disfunction in MMC, we can devise targeted therapies to further improve clinical outcomes. Future research may also lead to the development of biomarkers that can detect the presence and progression of fetal spinal cord injury in pregnancies affected by MMC. Such biomarkers could aid in the timing of prenatal intervention, likely a significant factor influencing neurological outcome. Ultimately, there remains a substantial need for novel strategies that can aid in the detection and management of MMC.

## Author Contributions

KJ, MM, GS, and BK contributed to the concept of the article, drafting, and editing of the manuscript. All authors approved the article for publication.

## Conflict of Interest

The authors declare that the research was conducted in the absence of any commercial or financial relationships that could be construed as a potential conflict of interest.

## References

[B1] Adzick N. S. (2012). Fetal surgery for myelomeningocele: trials and tribulations. Isabella forshall lecture. J. Pediatr. Surg. 47, 273–281. 10.1016/j.jpedsurg.2011.11.021 22325376 PMC3278714

[B2] Adzick N. S. Thom E. A. Spong C. Y. Brock J. W. III. Burrows P. K. Johnson M. P.. (2011). A randomized trial of prenatal versus postnatal repair of myelomeningocele. N. Engl. J. Med. 364, 993–1004. 10.1056/NEJMoa1014379 21306277 PMC3770179

[B3] Akalan N. (2011). Myelomeningocele (Open Spina Bifida)–Surgical Management. Adv. Tech. Stand. Neurosurg. 37, 113–141. 10.1007/978-3-7091-0673-0_5 21997743

[B4] Alles A. J. Sulik K. K. (1990). Retinoic acid-induced spina bifida: evidence for a pathogenetic mechanism. Development 108, 73–81. 2190788 10.1242/dev.108.Supplement.73

[B5] Belfort M. A. Whitehead W. E. Shamshirsaz A. A. Bateni Z. H. Olutoye O. O. Olutoye O. A.. (2017). Fetoscopic open neural tube defect repair: development and refinement of a two-port, carbon dioxide insufflation technique. Obstet. Gynecol. 129, 734–743. 10.1097/AOG.0000000000001941 28277363

[B6] Blumenfeld Y. J. Belfort M. A. (2018). Updates in fetal spina bifida repair. Curr. Opin. Obstet. Gyneco. 30, 123–129. 10.1097/GCO.0000000000000443 29489502

[B7] Borgstedt-Bakke J. H. Fenger-Gron M. Rasmussen M. M. (2017). Correlation of mortality with lesion level in patients with myelomeningocele: a population-based study. J. Neurosurg. Pediatr. 19, 227–231. 10.3171/2016.8.PEDS1654 27911247

[B8] Botelho R. D. Imada V. Rodrigues da Costa K. J. Watanabe L. C. Rossi Junior R. De Salles A. A. F.. (2017). Fetal myelomeningocele repair through a mini-hysterotomy. Fetal. Diagn. Ther. 42, 28–34. 10.1159/000449382 27656888

[B9] Botto L. D. Moore C. A. Khoury M. J. Erickson J. D. (1999). Neural-tube defects. N. Engl. J. Med. 341, 1509–1519. 10.1056/NEJM199911113412006 10559453

[B10] Bowman R. M. McLone D. G. Grant J. A. Tomita T. Ito J. A. (2001). Spina bifida outcome: a 25-year prospective. Pediatr. Neurosurg. 34, 114–120. 10.1159/000056005 11359098

[B11] Buffo A. Rite I. Tripathi P. Lepier A. Colak D. Horn A. P.. (2008). Origin and progeny of reactive gliosis: a source of multipotent cells in the injured brain. Proc. Natl. Acad. Sci. U S A 105, 3581–3586. 10.1073/pnas.0709002105 18299565 PMC2265175

[B12] Canfield M. A. Mai C. T. Wang Y. O’Halloran A. Marengo L. K. Olney R. S.. (2014). The association between race/ethnicity and major birth defects in the United States, 1999–2007. Am. J. Public Health 104, e14–e23. 10.2105/AJPH.2014.302098 25033129 PMC4151938

[B13] Ceccarelli G. Pozzo E. Scorletti F. Benedetti L. Cusella G. Ronzoni F. L.. (2015). Molecular signature of amniotic fluid derived stem cells in the fetal sheep model of myelomeningocele. J. Pediatr. Surg. 50, 1521–1527. 10.1016/j.jpedsurg.2015.04.014 26026346

[B14] Centers for Disease Control and Prevention (CDC). (2007). Hospital stays, hospital charges and in-hospital deaths among infants with selected birth defects—United States, 2003. MMWR. Morb. Mortal. Wkly. Rep. 56, 25–29. 17230142

[B15] Copp A. J. Greene N. D. Murdoch J. N. (2003). The genetic basis of mammalian neurulation. Nat. Rev. Genet. 4, 784–793. 10.1038/nrg1181 13679871

[B16] Correia-Pinto J. Tavares M. L. Baptista M. J. Henriques-Coelho T. Estevao-Costa J. Flake A. W.. (2002). Meconium dependence of bowel damage in gastroschisis. J. Pediatr. Surg. 37, 31–35. 10.1053/jpsu.2002.29422 11781982

[B17] Danzer E. Joyeux L. Flake A. W. Deprest J. (2020). Fetal surgical intervention for myelomeningocele: lessons learned, outcomes and future implications. Dev. Med. Child Neurol. 62, 417–425. 10.1111/dmcn.14429 31840814

[B18] Danzer E. Zhang L. Radu A. Bebbington M. W. Liechty K. W. Adzick N. S.. (2011). Amniotic fluid levels of glial fibrillary acidic protein in fetal rats with retinoic acid induced myelomeningocele: a potential marker for spinal cord injury. Am. J. Obstet. Gynecol. 204, 178.e1–178.e.11. 10.1016/j.ajog.2010.09.032 21284970

[B19] Dias M. S. McLone D. G. (1993). Hydrocephalus in the child with dysraphism. Neurosurg. Clin. N. Am. 4, 715–726. 10.1016/s1042-3680(18)30561-8 8241792

[B20] Dicianno B. E. Wilson R. (2010). Hospitalizations of adults with spina bifida and congenital spinal cord anomalies. Arch. Phys. Med. Rehabil. 91, 529–535. 10.1016/j.apmr.2009.11.023 20382283 PMC8474055

[B21] Dionigi B. Ahmed A. Brazzo J. III. Connors J. P. Zurakowski D. Fauza D. O. (2015a). Partial or complete coverage of experimental spina bifida by simple intra-amniotic injection of concentrated amniotic mesenchymal stem cells. J. Pediatr. Surg. 50, 69–73. 10.1016/j.jpedsurg.2014.10.004 25598096

[B22] Dionigi B. Brazzo J. A. III. Ahmed A. Feng C. Wu Y. Zurakowski D.. (2015b). Trans-amniotic stem cell therapy (TRASCET) minimizes Chiari-II malformation in experimental spina bifida. J. Pediatr. Surg. 50, 1037–1041. 10.1016/j.jpedsurg.2015.03.034 25929798

[B23] Drewek M. J. Bruner J. P. Whetsell W. O. Tulipan N. (1997). Quantitative analysis of the toxicity of human amniotic fluid to cultured rat spinal cord. Pediatr. Neurosurg. 27, 190–193. 10.1159/000121250 9577972

[B24] Farmer D. L. Thom E. A. Brock J. W. III. Burrows P.K. Johnson M. P. Howell L. J.. (2018). The management of myelomeningocele study: full cohort 30-month pediatric outcomes. Am. J. Obstet. Gynecol. 218, 256.e1–256.e13. 10.1016/j.ajog.2017.12.001 29246577 PMC7737375

[B25] Feng C. D Graham C. Connors J. P. Brazzo J. III. Zurakowski D. Fauza D. O. (2016). A comparison between placental and amniotic mesenchymal stem cells for transamniotic stem cell therapy (TRASCET) in experimental spina bifida. J. Pediatr. Surg. 51, 1010–1013. 10.1016/j.jpedsurg.2016.02.071 27013425

[B26] George T. M. Cummings T. J. (2003). The immunohistochemical profile of the myelomeningocele placode: is the placode normalw. Pediatr. Neurosurg. 39, 234–239. 10.1159/000072867 14512686

[B27] Heffez D. S. Aryanpur J. Hutchins G. M. Freeman J. M. (1990). The paralysis associated with myelomeningocele: clinical and experimental data implicating a preventable spinal cord injury. Neurosurgery 26, 987–992. 10.1097/00006123-199006000-00011 2362676

[B28] Heffez D. S. Aryanpur J. Rotellini N. A. Hutchins G. M. Freeman J. M. (1993). Intrauterine repair of experimental surgically created dysraphism. Neurosurgery 32, 1005–1010. 10.1227/00006123-199306000-00021 8327074

[B29] Heseker H. B. Mason J. B. Selhub J. Rosenberg I. H. Jacques P. F. (2009). Not all cases of neural-tube defect can be prevented by increasing the intake of folic acid. Br. J. Nutr. 102, 173–180. 10.1017/s0007114508149200 19079944

[B30] Housley H. T. Graf J. L. Lipshultz G. S. Calvano C. J. Harrison M. R. Farmer D. L.. (2000). Creation of myelomeningocele in the fetal rabbit. Fetal Diagn. Ther. 15, 275–279. 10.1159/000021021 10971080

[B31] Houtrow A. J. Thom E. A. Fletcher J. M. Burrows P. K. Adzick N. S. Thomas N. H. (2020). Prenatal repair of myelomeningocele and school-age functional outcomes. Pediatrics 145:e20191544 10.1542/peds.2019-1544 31980545 PMC6993457

[B34] Hunt G. M. (1990). Open spina bifida: outcome for a complete cohort treated unselectively and followed into adulthood. Dev. Med. Child Neurol. 32, 108–118. 10.1111/j.1469-8749.1990.tb16910.x 2186948

[B32] Hunt G. M. Oakeshott P. (2003). Outcome in people with open spina bifida at age 35: prospective community based cohort study. BMJ 326, 1365–1366. 10.1136/bmj.326.7403.1365 12816823 PMC162127

[B33] Hunt G. M. Poulton A. (1995). Open spina bifida: a complete cohort reviewed 25 years after closure. Dev. Med. Child Neurol. 37, 19–29. 10.1111/j.1469-8749.1995.tb11929.x 7828784

[B35] Hutchins G. M. Meuli M. Meuli-Simmen C. Jordan M. A. Heffez D. S. Blakemore K. J. (1996). Acquired spinal cord injury in human fetuses with myelomeningocele. Pediatr. Pathol. Lab. Med. 16, 701–712. 10.1080/713601226 9025869

[B36] Imbard A. Benoist J. F. Blom H. J. (2013). Neural tube defects, folic acid and methylation. Int. J. Environ. Res. Public Health 10, 4352–4389. 10.3390/ijerph10094352 24048206 PMC3799525

[B37] Johnson M. P. Bennett K. A. Rand L. Burrows P. K. Thom E. A. Howell L. J.. (2016). The management of myelomeningocele study: obstetrical outcomes and risk factors for obstetrical complications following prenatal surgery. Am. J. Obstet. Gynecol. 215, 778.e1–778.e9. 10.1016/j.ajog.2016.07.052 27496687 PMC5896767

[B38] Joyeux L. Danzer E. Flake A. W. Deprest J. (2018). Fetal surgery for spina bifida aperta. Arch. Dis. Child Fetal. Neonatal. Ed. 103, F589–F595. 10.1136/archdischild-2018-315143 30006470

[B39] Kaufman B. A. (2004). Neural tube defects. Pediatr. Clin. North Am. 51, 389–419. 10.1016/S0031-3955(03)00207-4 15062676

[B40] Keller-Peck C. R. Mullen R. J. (1997). Altered cell proliferation in the spinal cord of mouse neural tube mutants curly tail and Pax3 splotch-delayed. Dev. Brain Res. 102, 177–188. 10.1016/s0165-3806(97)00095-3 9352100

[B41] Kibar Z. Capra V. Gros P. (2007). Toward understanding the genetic basis of neural tube defects. Clin. Genet. 71, 295–310. 10.1111/j.1399-0004.2007.00793.x 17470131

[B42] Korenromp M. J. van Gool J. D. Bruinese H. W. Kriek R. (1986). Early fetal leg movements in myelomeningocele. Lancet 1, 917–918. 10.1016/s0140-6736(86)91022-6 2870386

[B43] Kowitzke B. Cohrs G. Leuschner I. Koch A. Synowitz M. Mehdorn H. M.. (2016). Cellular profiles and molecular mediators of lesion cascades in the placode in human open spinal neural tube defects. J. Neuropathol. Exp. Neurol. 75, 827–842. 10.1093/jnen/nlw057 27354486

[B44] Lazow S. P. Fauza D. O. (2020). Transamniotic stem cell therapy. Adv. Exp. Med. Biol. 1237, 61–74. 31302870 10.1007/5584_2019_416

[B45] Mazzone L. Pratsinis M. Pontiggia L. Reichmann E. Meuli M. (2016). Successful grafting of tissue-engineered fetal skin. Pediatr. Surg. Int. 32, 1177–1182. 10.1007/s00383-016-3977-z 27651371

[B46] Meuli M. Moehrlen U. (2014). Fetal surgery for myelomeningocele is effective: a critical look at the whys. Pediatr. Surg. Int. 30, 689–697. 10.1007/s00383-014-3524-8 24908159

[B47] Meuli M. Meuli-Simmen C. Hutchins G. M. Seller M. J. Harrison M. R. Adzick N. S. (1997). The spinal cord lesion in human fetuses with myelomeningocele: implications for fetal surgery. J. Pediatr. Surg. 32, 448–452. 10.1016/s0022-3468(97)90603-5 9094015

[B48] Meuli M. Meuli-Simmen C. Hutchins G. M. Yingling C. D. Hoffman K. M. Harrison M. R.. (1995a). *In utero* surgery rescues neurological function at birth in sheep with spina bifida. Nat. Med. 1, 342–347. 10.1038/nm0495-342 7585064

[B49] Meuli M. Meuli-Simmen C. Yingling C. D. Hutchins G. M. Hoffman K. M. Harrison M. R.. (1995b). Creation of myelomeningocele *in utero*: a model of functional damage from spinal cord exposure in fetal sheep. J. Pediatr. Surg. 30, 1028–1032; discussion 1032–1033. 10.1016/0022-3468(95)90335-6 7472926

[B50] Meuli M. Meuli-Simmen C. Yingling C. D. Hutchins G. M. Timmel G. B. Harrison M. R.. (1996). *In utero* repair of experimental myelomeningocele saves neurological function at birth. J. Pediatr. Surg. 31, 397–402. 10.1016/s0022-3468(96)90746-0 8708911

[B51] Mitchell L. E. Adzick N. S. Melchionne J. Pasquariello P. S. Sutton L. N. Whitehead A. S. (2004). Spina bifida. Lancet 364, 1885–1895. 10.1016/S0140-6736(04)17445-X 15555669

[B52] Moise K. J. Jr. Moldenhauer J. S. Bennett K. A. Goodnight W. Luks F. I. Emery S. P.. (2016). Current selection criteria and perioperative therapy used for fetal myelomeningocele surgery. Obstet. Gynecol. 127, 593–597. 10.1097/AOG.0000000000001296 26855109

[B54] Moldenhauer J. S. (2014). *In utero* repair of spina bifida. Am. J. Perinatol. 31, 595–604. 10.1055/s-0034-1372429 24819146

[B53] Moldenhauer J. S. Flake A. W. (2019). Open fetal surgery for neural tube defects. Best Pract. Res. Clin. Obstet. Gynaecol. 58, 58121–58132. 10.1016/j.bpobgyn.2019.03.004 31078425

[B55] Oakley G. P. Jr. (2010). Folic acid-preventable spina bifida: a good start but much to be done. Am. J. Prev. Med. 38, 569–570. 10.1016/j.amepre.2010.02.002 20409505

[B56] Oliver E. R. Heuer G. G. Thom E. A. Burrows P. K. Didier R. A. DeBari S. E. (2020). Myelomeningocele sac associated with worse lower extremity neurologic sequela: evidence for prenatal neural stretch injury? Ultra. Obstet. Gyn. 55, 740–746. 10.1002/uog.21891 31613408

[B57] Ornoy A. (2009). Valproic acid in pregnancy: how much are we endangering the embryo and fetus? Reprod. Toxicol. 28, 1–10. 10.1016/j.reprotox.2009.02.014 19490988

[B58] Osterhues A. Ali N. S. Michels K. B. (2013). The role of folic acid fortification in neural tube defects: a review. Crit. Rev. Food Sci. Nutr. 53, 1180–1190. 10.1080/10408398.2011.575966 24007422

[B59] Paek B. W. Farmer D. L. Wilkinson C. C. Albanese C. T. Peacock W. Harrison M. R.. (2000). Hindbrain herniation develops in surgically created myelomeningocele but is absent after repair in fetal lambs. Am. J. Obstet. Gynecol. 183, 1119–1123. 10.1067/mob.2000.108867 11084552

[B60] Parker S. E. Mai C. T. Canfield M. A. Rickard R. Wang Y. Meyer R. E.. (2010). Updated national birth prevalence estimates for selected birth defects in the United States, 2004–2006. Birth Defects Res. A. Clin. Mol. Teratol. 88, 1008–1016. 10.1002/bdra.20735 20878909

[B61] Pennington E. C. Gray F. L. Ahmed A. Zurakowski D. Fauza D. O. (2013). Targeted quantitative amniotic cell profiling: a potential diagnostic tool in the prenatal management of neural tube defects. J. Pediatr. Surg. 48, 1205–1210. 10.1016/j.jpedsurg.2013.03.009 23845608

[B62] Pennington E. C. Rialon K. L. Dionigi B. Ahmed A. Zurakowski D. Fauza D. O. (2015). The impact of gestational age on targeted amniotic cell profiling in experimental neural tube defects. Fetal Diagnosis. Ther. 37, 65–69. 10.1159/000362811 25171576

[B63] Perin L. Sedrakyan S. Da Sacco S. De Filippo R. (2008). Characterization of human amniotic fluid stem cells and their pluripotential capability. Methods Cell Biol. 86, 8685–8699. 10.1016/s0091-679x(08)00005-8 18442645

[B64] Radcliff E. Cassell C. H. Tanner J. P. Kirby R. S. Watkins S. Correia J.. (2012). Hospital use, associated costs and payer status for infants born with spina bifida. Birth Defects Res. A. Clin. Mol. Teratol. 94, 1044–1053. 10.1002/bdra.23084 23115108 PMC4507424

[B65] Reis J. L. Correia-Pinto J. Monteiro M. P. Hutchins G. M. (2007). *In utero* topographic analysis of astrocytes and neuronal cells in the spinal cord of mutant mice with myelomeningocele. J. Neurosurg. 106, 472–479. 10.3171/ped.2007.106.6.472 17566405

[B66] Roach J. W. Short B. F. Saltzman H. M. (2011). Adult consequences of spina bifida: a cohort study. Clin. Orthop. Relat. Res. 469, 1246–1252. 10.1007/s11999-010-1594-z 20878278 PMC3069297

[B67] Robel S. Berninger B. Gotz M. (2011). The stem cell potential of glia: lessons from reactive gliosis. Nat. Rev. Neurosci. 12, 88–104. 10.1038/nrn2978 21248788

[B68] Sandler A. D. (2010). Children with spina bifida: key clinical issues. Pediatr. Clin. North Am. 57, 879–892. 10.1016/j.pcl.2010.07.009 20883878

[B69] Sasso E. B. Thornton M. E. Chmait R. H. Ouzounian J. G. Grubbs B. H. (2020). Amylase concentration and activity in the amniotic fluid of fetal rats with retinoic acid induced myelomeningocele. J. Matern Fetal Neonatal. Med. 16, 1–8. 10.1080/14767058.2020.1713082 31910702

[B70] Selçuki M. Manning S. Bernfield M. (2001). The curly tail mouse model of human neural tube defects demonstrates normal spinal cord differentiation at the level of the meningomyelocele: implications for fetal surgery. Childs Nerv. Syst. 17, 19–23. 10.1007/s003810000401 11219618

[B71] Simpson J. L. Greene M. F. (2011). Fetal surgery for myelomeningocele? N. Engl. J. Med. 364, 1076–1077. 10.1056/NEJMe1101228 21306233

[B72] Sival D. A. Begeer J. H. Staal-Schreinemachers A. L. Vos-Niel J. M. Beekhuis J. R. Prechtl H. F. (1997). Perinatal motor behaviour and neurological outcome in spina bifida aperta. Early Hum. Dev. 50, 27–37. 10.1016/s0378-3782(97)00090-x 9467691

[B74] Stiefel D. Copp A. J. Meuli M. (2007). Fetal spina bifida in a mouse model: loss of neural function *in utero*. J. Neurosurg. 106, 213–221. 10.3171/ped.2007.106.3.213 17465388 PMC3651953

[B73] Stiefel D. Meuli M. (2007). Scanning electron microscopy of fetal murine myelomeningocele reveals growth and development of the spinal cord in early gestation and neural tissue destruction around birth. J. Pediatr. Surg. 42, 1561–1565. 10.1016/j.jpedsurg.2007.04.019 17848249

[B75] Sutton L. N. Sun P. Adzick N. S. (2001). Fetal neurosurgery. Neurosurgery 48, 124–142; discussion 142–144. 10.1097/00006123-200101000-00023 11152338

[B76] Tarui T. Kim A. Flake A. McClain L. Stratigis J. D. Fried I.. (2017). Amniotic fluid transcriptomics reflects novel disease mechanisms in fetuses with myelomeningocele. Am. J. Obstet. Gynecol. 217, 587.e1–587.e10. 10.1016/j.ajog.2017.07.022 28735706 PMC5671344

[B77] Tomlinson P. Sugarman I. D. (1995). Complications with shunts in adults with spina bifida. BMJ 311, 286–287. 10.1136/bmj.311.7000.286 7633231 PMC2550354

[B78] Turner C. G. Klein J. D. Wang J. Thakor D. Benedict D. Ahmed A.. (2013). The amniotic fluid as a source of neural stem cells in the setting of experimental neural tube defects. Stem Cells Dev. 22, 548–553. 10.1089/scd.2012.0215 22957979

[B79] Verhoef M. Barf H. A. Post M. W. van Asbeck F. W. Gooskens R. H. Prevo A. J. (2004). Secondary impairments in young adults with spina bifida. Dev. Med. Child Neurol. 46, 420–427. 10.1017/s0012162204000684 15174535

[B80] Viswanathan M. Treiman K. A. Kish-Doto J. Middleton J. C. Coker-Schwimmer E. J. Nicholson W. K. (2017). Folic acid supplementation for the prevention of neural tube defects: an updated evidence report and systematic review for the US preventive services task force. JAMA 317, 190–203. 10.1001/jama.2016.19193 28097361

[B81] Watanabe M. Jo J. Radu A. Kaneko M. Tabata Y. Flake A. W. (2010). A tissue engineering approach for prenatal closure of myelomeningocele with gelatin sponges incorporating basic fibroblast growth factor. Tissue Eng. Part A 16, 1645–1655. 10.1089/TEN.tea.2009.0532 19954327

[B82] Watanabe M. Kim A. G. Flake A. W. (2015). Tissue engineering strategies for fetal myelomeningocele repair in animal models. Fetal Diagn. Ther. 37, 197–205. 10.1159/000362931 25060746

[B83] Watanabe M. Li H. Kim A. G. Weilerstein A. Radu A. Davey M.. (2016). Complete tissue coverage achieved by scaffold-based tissue engineering in the fetal sheep model of Myelomeningocele. Biomaterials 76, 133–143. 10.1016/j.biomaterials.2015.10.051 26520044

[B84] Watanabe M. Li H. Roybal J. Santore M. Radu A. Jo J.. (2011). A tissue engineering approach for prenatal closure of myelomeningocele: comparison of gelatin sponge and microsphere scaffolds and bioactive protein coatings. Tissue Eng. Part A 17, 1099–1110. 10.1089/ten.TEA.2010.0390 21128864

[B85] Williams J. Mai C. T. Mulinare J. Isenburg J. Flood T. J. Ethen M.. (2015). Updated estimates of neural tube defects prevented by mandatory folic acid fortification-United States, 1995–2011. MMWR. Morb. Mortal. Wkly. Rep. 64, 1–5. 25590678 PMC4584791

[B86] Yu P. Wang H. Katagiri Y. Geller H. M. (2012). An *in vitro* model of reactive astrogliosis and its effect on neuronal growth. Methods Mol. Biol. 814, 327–340. 10.1007/978-1-61779-452-0_21 22144316 PMC4086427

[B87] Zieba J. Miller A. Gordiienko O. Smith G. M. Krynska B. (2017). Clusters of amniotic fluid cells and their associated early neuroepithelial markers in experimental myelomeningocele: correlation with astrogliosis. PLoS One 12:e0174625. 10.1371/journal.pone.0174625 28358903 PMC5373583

[B88] Zieba J. Walczak M. Gordiienko O. Gerstenhaber J. A. Smith G. M. Krynska B. (2019). Altered amniotic fluid levels of hyaluronic acid in fetal rats with myelomeningocele: understanding spinal cord injury. J. Neurotrauma 36, 1965–1973. 10.1089/neu.2018.5894 30284959 PMC7718849

